# IQSEC2-Associated Intellectual Disability and Autism

**DOI:** 10.3390/ijms20123038

**Published:** 2019-06-21

**Authors:** Nina S. Levy, George K. E. Umanah, Eli J. Rogers, Reem Jada, Orit Lache, Andrew P. Levy

**Affiliations:** 1 Technion Israel Institute of Technology, 1 Efron St., Haifa, 3525422, Israel; ninal@technion.ac.il (N.S.L.); eli.rogers@rochester.edu (E.J.R.); reemjada@campus.technion.ac.il (R.J.); eorit@technion.ac.il (O.L.); 2 Department of Neurology, Johns Hopkins University, Baltimore, MD 21205, USA; gumanah1@jhmi.edu

**Keywords:** intellectual disability, autism, AMPA receptors, NMDA receptors, guanine nucleotide exchange factor, synaptic plasticity

## Abstract

Mutations in *IQSEC2* cause intellectual disability (ID), which is often accompanied by seizures and autism. A number of studies have shown that IQSEC2 is an abundant protein in excitatory synapses and plays an important role in neuronal development as well as synaptic plasticity. Here, we review neuronal IQSEC2 signaling with emphasis on those aspects likely to be involved in autism. IQSEC2 is normally bound to N-methyl-D-aspartate (NMDA)-type glutamate receptors via post synaptic density protein 95 (PSD-95). Activation of NMDA receptors results in calcium ion influx and binding to calmodulin present on the IQSEC2 IQ domain. Calcium/calmodulin induces a conformational change in IQSEC2 leading to activation of the SEC7 catalytic domain. GTP is exchanged for GDP on ADP ribosylation factor 6 (ARF6). Activated ARF6 promotes downregulation of surface α-amino-3-hydroxy-5-methyl-4-isoxazolepropionic acid (AMPA)-type glutamate receptors through a c-jun N terminal kinase (JNK)-mediated pathway. NMDA receptors, AMPA receptors, and PSD-95 are all known to be adversely affected in autism. An *IQSEC2* transgenic mouse carrying a constitutively active mutation (A350V) shows autistic features and reduced levels of surface AMPA receptor subunit GluA2. Sec7 activity and AMPA receptor recycling are presented as two targets, which may respond to drug treatment in IQSEC2-associated ID and autism.

## 1. Introduction

This review will summarize our current knowledge of the molecular basis of intellectual disability (ID) in individuals with mutations in the *IQSEC2* gene and how compromised IQSEC2 function may be related to autism spectrum disorder (ASD). It is clear from clinical studies that autistic-like features are found in at least 25% of all *IQSEC2* ID cases [1,2,3]. This suggests a common biochemical pathway linking *IQSEC2*-associated ID and ASD. IQSEC2 is a guanine nucleotide exchange factor (GEF) that activates ADP ribosylation factor 6 (ARF6) and regulates proper membrane trafficking and synaptic structure and function in neurons. In this regard, ARF6 is important for maintaining the proper level of excitatory and inhibitory receptors essential for the normal learning process. Indeed, it is the imbalance between excitation and inhibition in synaptic transmission that has been proposed to underlie the pathophysiology of ASD. We propose a model by which IQSEC2 acts in promoting neurotransmission highlighting aspects which may be disrupted by mutations in *IQSEC2* resulting in ID and autism. 

## 2. Clinical Connection between *IQSEC2* and ASD

Mutations in the *IQSEC2* gene associated with ID are often accompanied by autism and/or epilepsy. In the first study establishing a linkage between *IQSEC2* and non-syndromic ID [1], four distinct missense mutations in *IQSEC2* were shown to segregate with affected individuals (57 total), with each family passing along one mutation. Autism was present in two of the families and among those two families, half of the affected individuals were autistic. Since that time, numerous reports documenting *IQSEC2* mutations have been published [2,3]. A recent review summarizing 136 individuals and 70 different types of mutations showed that autism is present in 25% of affected males and 30% of affected females [3]. Although *IQSEC2* is found on the X chromosome, in females, *IQSEC2* escapes X inactivation. This may explain the relatively high prevalence of both ID and autism in heterozygous females. Due to this high level of comorbidity, it seems likely that *IQSEC2*-associated ID and autism share common biochemical abnormalities.

## 3. IQSEC2 Structure and Function

**Figure 1:**
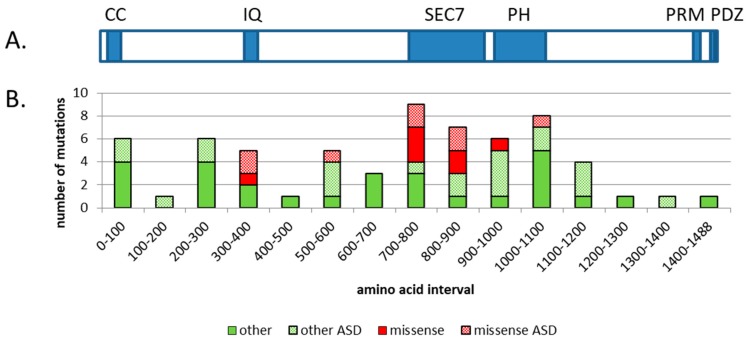
Schematic diagram of the conserved domains found in the human IQSEC2 protein and the distribution of mutations found in IQSEC2 cases. The top panel (A) depicts the protein encoded by the most abundant transcript for human *IQSEC2*, which contains 1488 amino acids. The known conserved domains and motifs are shown in blue. They are: CC-coiled coil, IQ–calmodulin binding site, SEC7-catalytic domain for GTP/GDP exchange on ARF, PH-pleckstrin homology domain, PRM–proline-rich motif, and PDZ-PSD-95 binding motif. The bottom panel (B) shows the distribution of mutations found in the *IQSEC2* gene (mutations were taken from Tables 2 and 3 from reference [3]) Missense mutations are shown in red; all other mutations (which include intragenic nonsense, duplication/truncation, in-frame deletions, and splicing variants) are shown in green. Hatched red bars show missense mutations associated with ASD. Hatched green bars show all other mutations associated with ASD. The positions of all mutations were arbitrarily chosen as the N terminal starting point. Mutations were considered to be associated with ASD if at least one member of the family was listed as having ASD traits or displaying autistic behavior.

IQSEC2 is named for two of its conserved regions known as the IQ (aa 347–376) and the SEC7 domains (aa 746–939) (see Figure 1). IQ stands for the amino acids, isoleucine and glutamine, which make up the beginning of this approximately 30-amino-acid domain that confers calcium calmodulin binding capacity to IQSEC2. The SEC7 domain, named for the original secretory mutant SEC7 from Saccharomyces cerevisiae, is approximately 200 amino acids and is responsible for the guanine nucleotide exchange function (GEF) of IQSEC2. The substrate for IQSEC2 GEF activity is thought to be ARF6. There are six mammalian ARFs, however ARF6 is the only one found associated with the plasma membrane and has been proposed to be the primary ARF for IQSEC2 [4]. ARF binds to two forms of the guanosine nucleotide, guanosine triphosphate (GTP) and guanosine diphosphate (GDP). IQSEC2 facilitates the exchange of GDP for GTP on ARF6 resulting in its activation. Additional functional domains in IQSEC2 include an N terminal coiled coil (CC) domain (aa 23–74) thought to promote self-assembly, a pleckstrin homology (PH) domain (aa 951–1085) that binds to phosphoinositides, and two C terminal binding motifs important for cytoskeletal organization: A proline-rich motif (PRM) (aa 1424–1434) and a PDZ binding motif (aa 1484–1488) (PDZ is an initialism combining the first letters of the first three proteins found to contain this common sequence: Post synaptic density protein (PSD95), Drosophila disc large tumor suppressor (Dlg1), and zonula occludens-1 protein (zo-1)).

Missense mutations in *IQSEC2* were concentrated in three functional domains including the IQ, SEC7, and PH domains (see Figure 1B) [3]. One missense mutation was found outside a known functional domain (R563N) and there was no ID associated with this case, only ASD traits. Other types of mutations, which mainly cause truncations or altered amino acid sequences, are scattered throughout the protein (see Figure 1 legend for details). Approximately half of all the mutations found in IQSEC2 were associated with ASD. The distribution of ASD-associated mutations across the gene was similar to non ASD mutations. This result indicates that mutations in *IQSEC2* that result in ASD cannot be attributed to any particular part of the protein. The reason that only half of the mutations in *IQSEC2* present with autism may be due to dosage effects or other variable pathology that occurs due to the stochastic nature of epileptic seizures.

An analysis of *IQSEC2* gene sequences from normal individuals has the potential to reveal mutations that do not cause ID and may be regarded as “tolerable”. This type of analysis found a large discrepancy between the predicted number of missense mutations (221) and those that were observed (86) [3]. This discrepancy may be due to the fact that missense mutations in regions other than the known functional domains do cause pathology, albeit less severe than that seen in IQSEC2-associated ID. Perhaps these cases are more mild forms of ID and or ASD with no epilepsy, which is the primary reason for doing exome sequencing of IQSEC2, yet not mild enough to be included in a normal group. An example might be the R563N mutation mentioned above. A previous study of mutation intolerance showed that genes involved in early onset neurodevelopmental disorders such as the epileptic encephalopathies carry the highest level of mutational intolerance [5]. One possibility may be that genes in this class are often found in large complexes with scaffolding proteins and other proteins involved in signal transduction. The architecture and dynamics of these complexes may be so intricate that most amino acid changes would alter their proper function. 

## 4. IQSEC2 and Spine Formation

In the brain, where IQSEC2 is predominantly expressed, it has been shown that ARF6 is important in the regulation of dendritic spine development [6]. During embryonic and neonatal growth, neuronal dendrites lengthen and become extensively branched. This process increases their surface area and allows for more connections between neurons. Maturing dendrites develop small protrusions called dendritic spines, which are the site of the majority of the excitatory synapses in the brain. Spines begin as long, thin filopodia and develop into large, mature spines with a defined spine head containing neurotransmitter receptors and a postsynaptic density (PSD). The PSD is an electron-dense region attached to the postsynaptic membrane. The PSD is in close apposition to the presynaptic membrane and ensures that receptors are in close proximity to presynaptic neurotransmitter release sites. Many proteins in the PSD are involved in the regulation of synaptic function. Key among these are postsynaptic density-95 (PSD95), NMDA receptors, AMPA receptors, calcium/calmodulin-dependent protein kinase II, and actin. 

During the first few years of life, there is an intense increase in spine density, which is followed by a gradual decline in the number of spines, known as spine pruning. The elimination of spines occurs from childhood to adulthood and is the time of fine structural reorganization of the cortex. This period is associated with higher cognitive function such as learning and memory. This process is characterized by synaptic plasticity, whereby a neuron changes its synaptic strength based on previous stimuli. Synaptic strength is the average amount of current produced in the postsynaptic neuron by an action potential in the presynaptic neuron. Neurons may undergo long-term potentiation (LTP), or a sustained increase in synaptic strength. In contrast, neurons may decrease their synaptic strength for a prolonged period of time, otherwise known as long-term depression (LTD). The size of the dendritic spines and their PSDs have been observed to increase with LTP and decrease with LTD [7].

IQSEC2 has been localized to dendritic spines by immunocytochemistry of cultured rat and mouse hippocampal neurons [4,8]. The importance of IQSEC2 in spine development has been shown in studies in which knocking down IQSEC2 mRNA levels in mouse primary hippocampal cell cultures disrupted dendritic spine morphogenesis [9]. Specifically, there was an increase in the density of dendritic spines after two weeks in culture compared to controls with no change in the maturity of the spines. Overexpression of wild-type IQSEC2 in similar cultures led to a decrease in density of dendritic spines but an increase in spine maturity. IQSEC2 knockout mice gave rise to neuronal cultures that were more disorganized than their wildtype littermates. Taken together, these data suggest that the level of IQSEC2 is critical for normal spine development and maturation.

## 5. Proteins Implicated in ASD that Interact with IQSEC2

### 5.1. PSD-95 

The prominence of IQSEC2 in neuronal spine formation makes it a logical candidate in affecting autism. This is because a major cellular phenotype identified in autism patients is dendritic spine aberrations. Normally, during development spine pruning occurs as part of the spinal maturation process. In brains from autistic patients, there is more rapid growth and formation during childhood and less spinal pruning and elimination during adulthood. This leads to an overabundance of spines in ASD and results in increased spine density and hyper connectivity [10,11]. Interestingly, there seems to be somewhat of an opposite effect in ID, where spines on cortical neurons from individuals with intellectual disability were described as having an immature appearance with fewer spines per dendritic branch. The dendrites of cortical neurons from ID patients have been reported to be less complex than normal subjects [11]. *IQSEC2*-associated ID is likely to be a very specific subclass of all ID subjects and may not be represented by the above results. Further studies on mouse models or induced pluripotent stem cells (IPSC) cells will likely clarify this issue.

One of the most abundant proteins in the PSD is PSD-95. It is a member of the membrane-associated guanylate kinase (MAGUK) family and functions as a scaffolding protein, anchoring receptor molecules in the membrane to the cytoskeleton. PSD-95 contains three PDZ domains, a common structural domain of 80–90 amino-acids that plays a key role in holding together and organizing signaling complexes at cellular membranes. There is compelling evidence that mutations in PSD-95 result in cognitive and learning deficits associated with autism and schizophrenia [10]. PSD-95 is present in the PSD in complexes as large as 1.5 MDa [12]. A subset of these complexes contains IQSEC2. In mice lacking PSD-95, IQSEC2 does not form 1.5 MDa complexes [13]. Separating and identifying the members of PSD-95/IQSEC2 super complexes will likely shed new light on the mechanism of IQSEC2 signaling. 

IQSEC2 has been shown to associate with PSD-95 in the post synaptic density on excitatory synapses [8]. This binding is thought to occur via a four-amino-acid sequence STVV in the C terminus of IQSEC2. A patient with a frameshift mutation in the C terminal region of IQSEC2 quite close to the PDZ binding motif (G1468A) supports the crucial role of this domain [3]. In addition, mutations in *IQSEC2’s* PDZ domain were found to disrupt glutamate receptor trafficking and neurotransmission in organotypic hippocampal cultures [14], as well as IQSEC2 localization to dendritic spines [15], as will be discussed below.

### 5.2. IRSp53/BAIAP2, PSD-93, SAP97, CaMKIIa

Insulin receptor substrate of 53 kDa (IRSp53), also known as brain-specific angiogenesis inhibitor 1-associated protein 2 (BAIAP2), is a multidomain scaffolding protein that is present in high levels in the PSD at excitatory synapses and regulates actin dynamics at dendritic spines [16]. IRSp53 has also been found in connection with ASD as well as other behavioral disorders. Mice that lack IRSp53 show increased NMDA receptor function and display cognitive deficits. Drug treatment that suppresses NMDA receptor activation alleviates some of these deficits. IRSp53 knockout mice display similar behaviors to *IQSEC2* A350V mutant mice [17] such as hyperactivity in open field locomotion, normal rotarod motor function, impaired Morris water maze memory, and decreased three-chamber social interaction [16].

It has been reported that the PRM region of IQSEC2 (see Figure 1) interacts with IRSp53, as evidenced by co-immunoprecipitation experiments [15]. Deletion of the 74 C-terminal amino acids of IQSEC2 prevented localization of IQSEC2 to dendritic spines versus dendritic shafts. Deletion of the last four amino acids (the PDZ binding motif) did not prevent spine localization. A separate mutation of the PRM also did not prevent spine localization, indicating a potential redundancy in this particular function by the PRM and the PDZ binding motifs. Alternatively, there may be critical sequences outside these two domains in the C terminus that are necessary for spine localization.

Other proteins that have been shown to be associated with IQSEC2 by co-immunoprecipitation are PSD93, synapse-associated protein 97 (SAP97), and calcium calmodulin kinase IIa (CaMKIIa) [4]. SAP97 and PSD-93 are members of the MAGUK family of scaffold proteins and contain PDZ domains. PSD-93, along with PSD-95 and the NMDA receptor subunit GluN2B, were found to be essential for the formation of 1.5 MDa NMDA supercomplexes [12]. As mentioned above, IQSEC2 is a member of a subset of these complexes. CaMKII is also present at very high levels in the PSD and is thought to be an important mediator of learning and memory. Aside from its phosphorylation function, CaMKII has protein docking capability. Specifically, CaMKII is a central organizer of the postsynaptic F-actin network and can autoregulate its position in the PSD while binding to other effector proteins [18] It is becoming clear that understanding IQSEC2 signal transduction will require teasing apart the biochemical composition and dynamics of the large PSD complexes.

### 5.3. Glutamate Receptors

IQSEC2 is as abundant as many of the NMDA and AMPA glutamate receptors in the PSD of glutamatergic synapses [19]. These two receptor types are responsible for mediating excitatory neurotransmission and neuronal plasticity, the process by which neurons acquire a long-term change in their excitability based on previous stimuli. Neuronal synaptic plasticity is critical for learning and memory. Disruption of these pathways leads to neurodevelopmental diseases, including ID and autism. A recent review summarizes the disturbances in AMPA/NMDA receptor expression and function for 31 different synaptic genes implicated in autism [20]. In two well-known mouse models of autism, it was shown that expression of AMPA receptors was disturbed [21].

The importance of IQSEC2 in glutamate receptor function was shown using organotypic rat hippocampal slices. Transfection of normal IQSEC2 resulted in decreased electrophysiological responses characteristic of AMPA receptors [22]. There was no change in NMDA receptor response. Transfection of an N terminal deletion construct (213 aa), which removes the coiled coil domain, resulted in an increased AMPA receptor response, possibly due to a dominant negative effect. Blocking of NMDA receptors reversed the effects of wild-type and N terminally deleted IQSEC2, indicating that spontaneous synaptic activity activates NMDA receptors, which, in turn, activates IQSEC2, producing a decrease in AMPA receptors. This decrease was shown to be dependent on SEC7 activity. Transfection of the IQSEC2 construct BRAG-IQ containing a mutated IQ region (rendering it unable to bind to calmodulin) resulted in a decreased AMPA receptor response that was not dependent on NMDA receptor signaling. The authors suggest that the BRAG-IQ mutant is constitutively active and does not require calcium-induced release of calmodulin to undergo an activating conformational change [22].

A second group studying synaptic transmission found that transfection of wild-type *IQSEC2* into organotypic hippocampal neurons resulted in an increase in AMPA receptor signaling that was independent of SEC7 activity but required the presence of the PDZ binding motif [14]. In addition, surface expression of GluA2 was shown to be increased by IQSEC2. The increase in AMPA signaling in this study is opposite to that discussed above [22] where a decrease in AMPA signaling was seen with wild-type IQSEC2. Differences in the age of the cell cultures used by both groups may be an important factor in resolving the discrepant results. It has been shown that during postnatal weeks 2–3, there is a switch from NMDA receptor subunit GluN2B-IQSEC2 signaling to GluN2A-IQSEC1 signaling in cortical cultures [23]. These effects were also seen in hippocampal neurons and may be responsible for the differences seen above.

Additional experiments by the second group [14] involved induction of long-term potentiation (LTP) and long-term depression (LTD) in *IQSEC2*-transfected organotypic hippocampal slices. The results showed that IQSEC2 was not involved in LTP, whereas LTD was dependent on IQSEC2 containing a functional SEC7 domain and the C terminal PDZ binding motif. In addition, immunoprecipitation experiments showed that in addition to PSD-95, IQSEC2 was found in complexes containing the NR1 and NR2A subunits of NMDA receptors. These results are in agreement with the finding that IQSEC2 plays a role in AMPA receptor removal mediated by NMDA receptor activity [22]. 

A recent study has been conducted using a transgenic mouse model of *IQSEC2* carrying a mutation in the IQ region (A350V) originally found in a young male subject with moderate to severe ID and ASD [24]. The mice display seizures between days 14–21 which results in an approximately 40% mortality rate among hemizygous males and 20% mortality among heterozygous females [17]. Biochemical analysis revealed that transgenic mice carrying the A350V mutation displayed decreased levels of surface AMPA receptor subunit GluA2, as assessed by FACS analysis of hippocampal cells, immunocytochemistry of hippocampal brain slices, and immunoblotting of crosslinked cell surface proteins from hippocampi [17]. This mutation was associated with a calcium-independent GEF activity as assessed by the GGA3 pull down assay in cells treated with or without ionomycin, similar to the BRAG1-IQ mutation discussed above [22].

It was further shown that wild-type IQSEC2 does bind to calcium/calmodulin as measured by binding to calmodulin sepharose, with half maximal binding occurring at 1 μM [17]. The A350V mutant protein also bound to calcium/calmodulin sepharose with similar affinity. However, when measured in HEK cells using the Lumier assay, the A350V mutant IQSEC2 bound much less efficiently to apocalmodulin when compared to wild-type IQSEC2. Discrepant results stating that calcium releases calmodulin bound to IQSEC2 [22] may have been due to different concentrations of Triton X-100 present in the lysis buffer used by the two groups.

**Figure 2:**
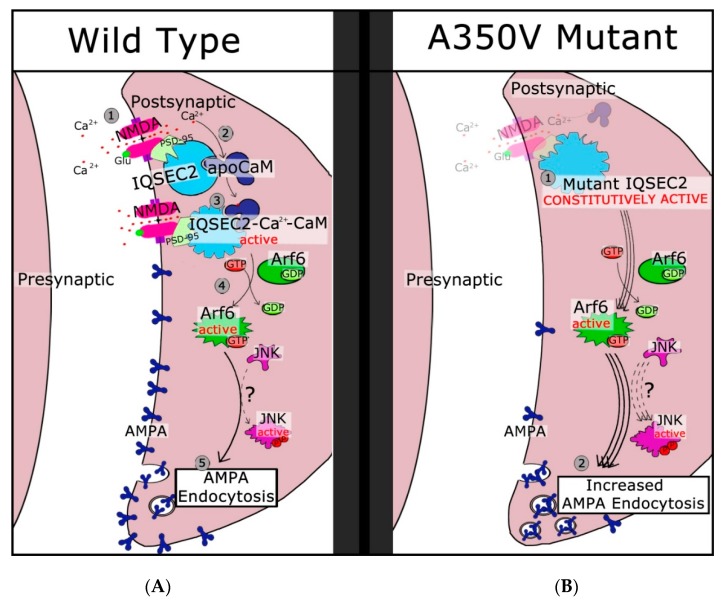
Signal transduction pathway in wild type and A350V mutant IQSEC2. In the left panel (**A**), binding of glutamine to the NMDA receptor (pink shape) allows calcium ion influx (step 1). Binding of calcium to calmodulin present on IQSEC2 (light blue shape) causes a conformational change (step 2) in IQSEC2 resulting in activation of the SEC7 catalytic domain (step 3). GDP is exchanged for GTP on ARF6 (green shape), resulting in active ARF6 (step 4). Additional steps including JNK activation (purple shape) lead to endocytosis of AMPA receptors (step 5). In the right panel (**B**), the A350V mutant IQSEC2 is constitutively active (step 1) leading to constant activation of ARF6 and JNK, enhanced endocytosis of AMPA receptors (step 2), and decreased surface AMPA expression. (Reproduced from reference 17.)

In summary, we can suggest that the initial steps in IQSEC2 signaling are as follows (see Figure 2): IQSEC2 is normally bound to apocalmodulin in the cell under basal conditions. IQSEC2/calmodulin is found in complex with PSD-95 and NMDA receptors and likely a large number of additional proteins. When glutamate binds to the NMDA receptor, it becomes activated, allowing an influx of calcium, which binds to calmodulin present on IQSEC2 and causes a conformational change. This alteration in protein conformation activates the SEC7 domain and leads to an increase in ARF-GTP. In the case of the A350V mutant, IQSEC2 adopts a constitutively active conformation, leading to increased ARF6-GTP and a decrease in AMPA receptors. The steps leading from active ARF-GTP to changes in surface AMPA receptors are less well understood but appear to involve JNK kinase [22]. 

## 6. Therapeutic Treatment of IQSEC2-Associated ID and Autism

The above data suggest possible targets for drug treatment in *IQSEC2*-associated ID and autism based on the molecular pathophysiology associated with specific mutations. One potential target is the Sec7 activity of IQSEC2. A second potential target is the regulation of AMPA receptor recycling. With regards to the constitutively elevated Sec7 activity demonstrated by the A350V mutation, the objective would be to restore the normal situation wherein the Sec7 activity is normally inactive and is only induced by calcium influx through the NMDA receptor. A precise personalized medicine for this mutation would be a drug that was specific for the Sec7 domain of A350V IQSEC2 and whose activity would itself be inhibited by intracellular calcium. We are currently working on designing such a drug. For the second target, the recycling of AMPA receptors, several drugs are currently in existence [25]. Specifically, the drug parampanel inhibits recycling, while ritalin and aniracetam increase recycling. The recently described positive allosteric modulators (PAM) of AMPA receptor activity such as PF-4778574 may serve to increase AMPAR activity in the setting of an absolute decrease in AMPAR levels.

The importance of understanding the precise mechanism underlying a given mutation for personalized therapy is underscored by the apparent difference in the molecular pathophysiology of different *IQSEC2* mutations. The work of a number of investigators [14,22] proposes that many IQSEC2 mutations (specifically truncations or missense mutations in Sec7) result in a down regulation of IQSEC2 Sec7 activity and a corresponding increase in surface AMPAR. This precisely represents the opposite pathophysiology of what is seen with A350V *IQSEC2*. A similar paradigm whereby two different mutations in the same gene can oppositely regulate AMPA receptors was recently demonstrated for the thorase gene where some mutations decrease thorase activity and are associated with an increase in AMPA receptors (treatable with parampanel) while other mutations increase thorase activity and they are associated with a decrease in surface AMPA receptors, which might be treatable with AMPA receptor PAMS [26,27]. Transgenic mice carrying *IQSEC2* mutations as well as pluripotent stem cells derived from patient tissue may serve as models for testing these approaches to treat specific *IQSEC2* mutations associated with ID and autism. 

## Abbreviations

ID	Intellectual disability
NMDA	N-methyl-D-aspartate
PSD	Post synaptic density
GTP	Guanosine triphosphate
GDP	Guanosine diphosphate
ARF	ADP ribosylation factor
AMPA	α-Amino-3-hydroxy-5-methyl-4-isoxazolepropionic acid
GABA	Gamma-aminobutyric acid
ASD	Autism spectrum disorder
CC	Coiled coil
PH	Pleckstrin homology
PRM	Proline rich motif
PDZ	Post synaptic density protein (PSD95), Drosophila disc large tumor suppressor (Dlg1), and zonula occludens-1 protein (zo-1)
GEF	Guanine nucleotide exchange factor
MAGUK	Membrane associated guanylate kinase
IRSp53	Insulin receptor substrate of 53 kda
BAIAP2	Brain specific angiogenesis inhibitor 1-associated protein 2
SAP97	Synapse associated protein 97
CaMKIIa	Calcium calmodulin kinase iia
GGA3	Golgi associated gamma adaptin ear containing ARF binding protein 3
JNK	C-jun N terminal kinase
PAM	Positive allosteric modulators
